# RsfA (YbeB) Proteins Are Conserved Ribosomal Silencing Factors

**DOI:** 10.1371/journal.pgen.1002815

**Published:** 2012-07-19

**Authors:** Roman Häuser, Markus Pech, Jaroslaw Kijek, Hiroshi Yamamoto, Björn Titz, Florian Naeve, Andrey Tovchigrechko, Kaori Yamamoto, Witold Szaflarski, Nono Takeuchi, Thorsten Stellberger, Markus E. Diefenbacher, Knud H. Nierhaus, Peter Uetz

**Affiliations:** 1 Institute of Toxicology and Genetics, Karlsruhe Institute of Technology (KIT), Karlsruhe, Germany; 2 German Cancer Research Center (DKFZ), Heidelberg, Germany; 3 Abteilung Vingron, AG Ribosomen Max-Planck-Institut für Molekulare Genetik, Berlin, Germany; 4 Institut für Medizinische Physik und Biophysik, Charité–Universitätsmedizin Berlin, Berlin, Germany; 5 J. Craig Venter Institute (JCVI), Rockville, Maryland, United States of America; 6 Department of Histology and Embryology, Poznan University of Medical Sciences, Poznan, Poland; 7 Department of Medical Genome Sciences, Graduate School of Frontier Sciences, University of Tokyo, Kashiwa-shi, Chiba, Japan; 8 Proteros Biostructures, Martinsried, Germany; 9 Center for the Study of Biological Complexity, Virginia Commonwealth University, Richmond, Virginia, United States of America; Uppsala University, Sweden

## Abstract

The YbeB (DUF143) family of uncharacterized proteins is encoded by almost all bacterial and eukaryotic genomes but not archaea. While they have been shown to be associated with ribosomes, their molecular function remains unclear. Here we show that YbeB is a ribosomal silencing factor (RsfA) in the stationary growth phase and during the transition from rich to poor media. A knock-out of the *rsfA* gene shows two strong phenotypes: (i) the viability of the mutant cells are sharply impaired during stationary phase (as shown by viability competition assays), and (ii) during transition from rich to poor media the mutant cells adapt slowly and show a growth block of more than 10 hours (as shown by growth competition assays). RsfA silences translation by binding to the L14 protein of the large ribosomal subunit and, as a consequence, impairs subunit joining (as shown by molecular modeling, reporter gene analysis, *in vitro* translation assays, and sucrose gradient analysis). This particular interaction is conserved in all species tested, including *Escherichia coli, Treponema pallidum, Streptococcus pneumoniae, Synechocystis PCC 6803*, as well as human mitochondria and maize chloroplasts (as demonstrated by yeast two-hybrid tests, pull-downs, and mutagenesis). RsfA is unrelated to the eukaryotic ribosomal anti-association/60S-assembly factor eIF6, which also binds to L14, and is the first such factor in bacteria and organelles. RsfA helps cells to adapt to slow-growth/stationary phase conditions by down-regulating protein synthesis, one of the most energy-consuming processes in both bacterial and eukaryotic cells.

## Author Summary

The YbeB/DUF143 family of proteins is one of the most widely conserved proteins with homologues present in almost all bacteria and eukaryotic organelles such as mitochondria and chloroplasts (but not archaea). While it has been shown that these proteins associate with ribosomes, their molecular function remained mysterious. Here we show that a knock-out of the *ybeB* gene causes a dramatic adaptation block during a shift from rich to poor media and seriously deteriorates the viability during stationary phase. YbeB of six different species binds to ribosomal protein L14. This interaction blocks the association of the two ribosomal subunits and, as a consequence, translation. YbeB is thus renamed “RsfA” (ribosomal silencing factor A). RsfA inhibits translation when nutrients are depleted (or when cells are in stationary phase), which helps the cell to save energy and nutrients, a critical function for all cells that are regularly struggling with limited resources.

## Introduction

*Escherichia coli* harbors a core set of about 190 genes that are conserved in more than 90% of all completely sequenced genomes [1]. Most of them encode well-understood proteins involved in metabolism, transcription, translation, or replication. However, a few of these highly conserved proteins remain functionally uncharacterized and thus enigmatic. One of these mysterious proteins is YbeB. In 2004 it was proposed by Galperin and Koonin as one of 10 top targets of conserved hypothetical proteins for experimental characterization [2]. In recent interactome studies, we and others found this protein to interact with various proteins, including several ribosomal components [3], [4], [5], [6]. Moreover, YbeB was shown to co-sediment with the large ribosomal subunit (LRS) [7], suggesting that it functions in protein translation. Recently it has been suggested that its mitochondrial homologue, C7orf30, is involved in ribosome biogenesis and/or translation [5], [8] although these studies were not able to explain their observations mechanistically. In this work we characterize YbeB's molecular function by identifying its binding site on the LRS and reveal a molecular mechanism of YbeB action: it is down-regulating protein synthesis under nutrient shortage by binding to protein L14 of the LRS, acting as a ribosomal silencing factor (“RsfA”) by blocking ribosome subunit joining. Thus, we will use the term “RsfA” below.

## Results

### RsfA homologues are conserved from bacteria to humans and interact with the ribosomal protein L14

**Figure 1:**
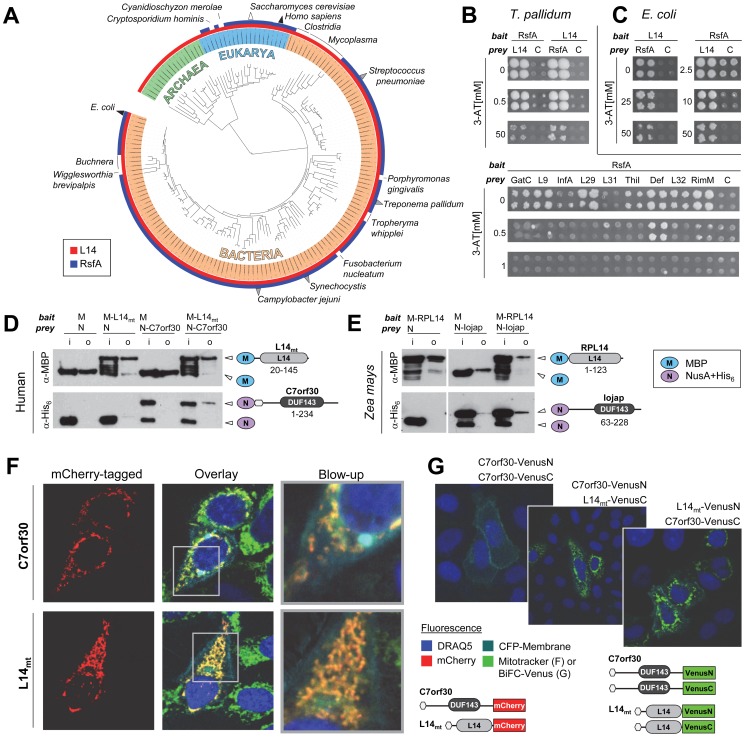
RsfA and L14 and their interaction are conserved in bacteria and eukaryotic organelles. (A) Phylogenetic distribution of RsfA (Interpro entry IPR004394 [DUF143]) and ribosomal protein L14 (IPR000218) on the iTOL tree of life [59]. Triangles indicate species in which the RsfA-L14 interaction was detected by binary detection assays (grey), co-purification with the LRS (white) or both (black). Known RsfA-L14/LRS interactions are listed in Table S1. (B) *T. pallidum* RsfA (TP0738) interacts strongly with L14 (TP0199) and very weakly with other proteins involved in translation [6] in yeast-two-hybrid assays. C, control (with empty prey vector to measure self-activation of the bait). This interaction is also conserved in *E. coli* (C). (D, E) RsfA and L14 homologues from human and maize interact in pull down experiments. RsfA homologues were tagged with NusA-His_6_ (N) and L14 homologues with maltose binding protein (M) (human mtRsfA = C7orf30, mitochondrial ribosomal protein L14 = L14_mt_; maize RsfA = Iojap, maize chloroplastic L14 = RPL14); i = input samples, o = output samples. Constructs with the corresponding Interpro signatures and the range of cloned codons are illustrated on the right. (F) Human mitochondrial C7orf30 (mtRsfA) co-localizes with L14_mt_ exclusively into mitochondria as visualized by MitoTracker Green. Nuclei visualized by DRAQ5 (blue) and membranes by eCFP-membrane (cyan). Co-localization of both mtRsfA (C7orf30) and L14_mt_ in mitochondria is indicated in yellow. (G) Bi-molecular fluorescence complementation (BiFC) reveals the interaction of mtRsfA (C7orf30) and L14_mt_ in mitochondria. Overlay images represent DRAQ5 (blue), CFP-membrane (cyan) and BiFC stained cells. Green fluorescence indicates interaction-dependent regeneration of the Venus protein. Constructs are shown below. Here, the hexagons symbolize the native N-termini including mitochondrial localization sequences.

In the Pfam database (V26.0) RsfA sequence homologues are known for at least 2,928 species, including nearly all bacteria as well as almost all eukaryotic species (Pfam entry PF02410, Interpro IPR004394). However, the RsfA protein family is conspicuously absent in archaea (Figure 1A). In the STRING 9.0 database [9] RsfA is clustered with the orthologous protein group “COG0799”, consisting of 932 RsfA homologues in 920 different species, indicating that there is usually one *rsfA* gene per genome. A multiple sequence alignment of ten representative RsfA orthologues, however, exhibits only limited conservation when compared to ribosomal protein L14 (Figure S1).

Interestingly, more than 80% of all eukaryotic RsfA orthologues are predicted to localize to mitochondria or chloroplasts according to the WoLF PSort program [10]. For the yeast orthologue ATP25, the mitochondrial localization has been experimentally confirmed [11] and the *Zea mays* homologue, Iojap, was found in chloroplast fractions [12]. This strongly suggests that RsfA functions in a strictly conserved process of bacterial origin. Previously, Butland and colleagues reported L14, L19, L4, L7/L12 and others as interaction partners of RsfA based on protein complex data [3]. Similarly, we found that several interactors of RsfA's *Treponema pallidum* orthologue TP0738 were involved in protein synthesis [6]. Although these observations provided the first experimental hint that RsfA might function in translation, this has never been functionally demonstrated. Since previous studies have revealed RsfA's association with the large ribosomal subunit (LRS) which offers multiple binding sites, we re-tested all previously detected interactions of *T. pallidum* RsfA that are involved in protein translation. As expected, several proteins indeed tested positive (Figure 1B). However, the interaction of RsfA with L14 was by far the strongest as determined by using increasing concentrations of 3-amino-triazole (3-AT), a competitive inhibitor of the yeast two-hybrid reporter gene *HIS3*. In fact, only the interaction with L14 was detectable at more than 1 mM 3-AT. Furthermore, the L14-RsfA interaction was the only one that was detectable in a reciprocal screen, *i.e.* with RsfA used as both bait and prey.

Given the conservation of RsfA, we wanted to establish to which extent the interactions of RsfA of *T. pallidum* are conserved in other species. To this end, we first retested whether the interactions of *T. pallidum* RsfA are conserved in *E. coli*. We also included eight putative interaction partners that have been identified in a protein complex together with *E. coli* RsfA and L14 [3] and four interologous pairs detected by Y2H in *Campylobacter jejuni* [13]. Surprisingly, only the interaction with L14 was conserved in *E. coli* as a strong (up to 50 mM 3-AT) and reciprocal interaction (Figure 1C, all tested interactions and reference sets are listed in Table S2 and the complete Y2H assays are shown in Figure S2). Moreover, we confirm the interaction of RsfA with L14 from *E. coli* independently in a pull-down experiment (Figure S3A).

Thus, we conclude that L14 is the primary and specific binding target of RsfA on the LRS and that all other interactions are species specific or even artifacts.

Next we tested whether this particular interaction is conserved in other bacteria. Notably, we could verify the interaction in all tested species, including gram-positive *Streptococcus pneumoniae* and the cyanobacterium *Synechocystis PCC 6803* (Figure S3B and S3C). In addition, we confirmed the interaction between the corresponding orthologues of RsfA/L14 of both human (C7orf30/mitochondrial L14) and *Zea mays* (Iojap/chloroplastic RPL14) as shown in Figure 1D and 1E, respectively.

In HeLa cells human C7orf30 co-localized with L14_mt_ exclusively to mitochondria (Figure 1F). This supports the hypothesis that eukaryotic RsfA orthologues are functionally active only in organelles. Finally, we verified the human protein interaction *in vivo* by a bimolecular fluorescence complementation assay using C-terminally tagged Split-Venus constructs (Figure 1G). In summary, these results strongly suggest that the interaction of RsfA and L14 is universally conserved in all species that encode RsfA homologues and that in fact their specific binding site at the LRS is in the ribosomal protein L14.

### RsfA binds to critical residues of ribosomal protein L14 at the ribosomal subunit interface

**Figure 2:**
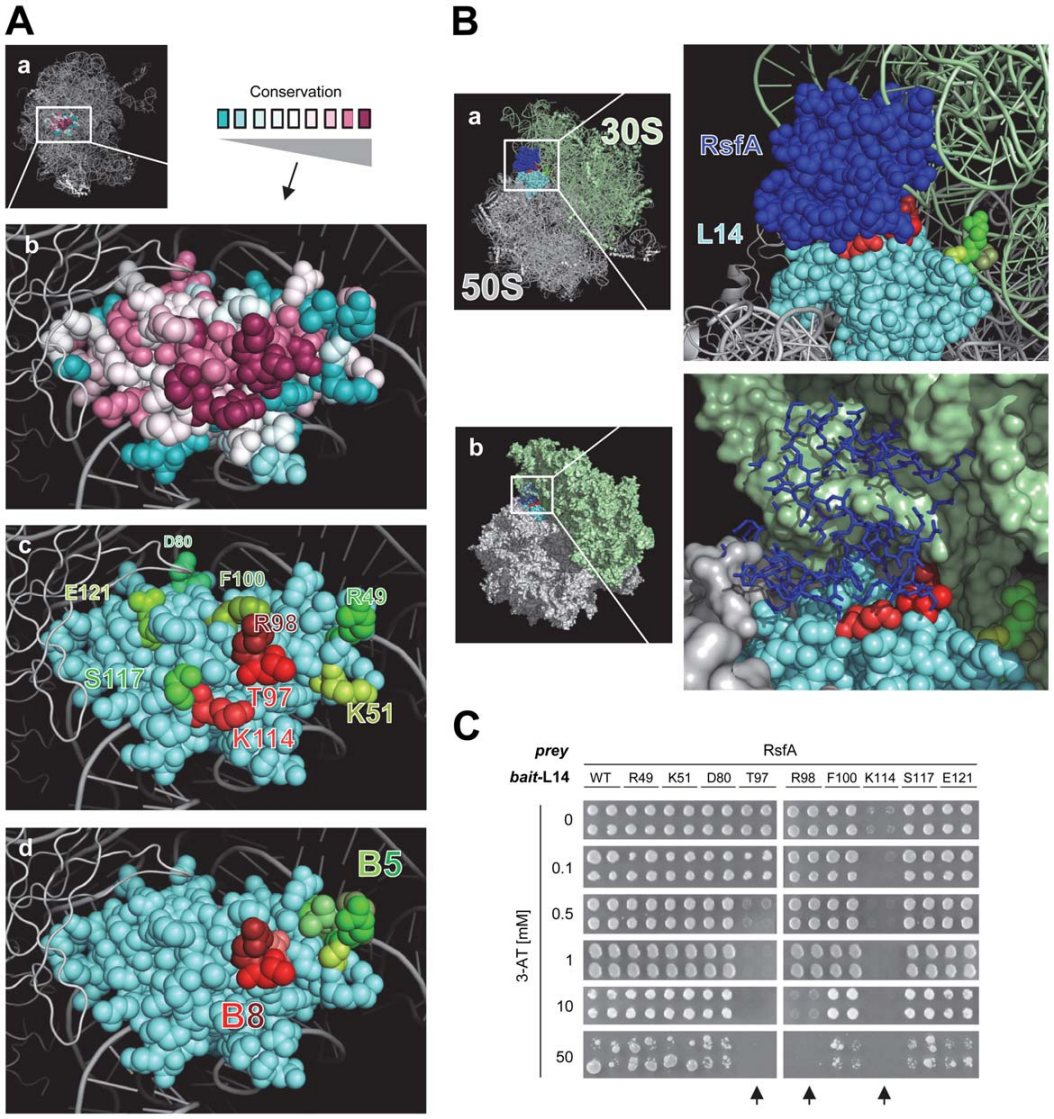
Mapping the RsfA binding site on ribosomal protein L14. (A) L14 in the context of the 3D structure of the 50S ribosomal subunit (a) (PDB: 2AWB) [14]. (b) Conserved residues of L14: magenta (highly conserved), grey (moderately conserved), turquoise (little or no conservation). (c) Mutated residues for interaction epitope mapping (red or green); residues involved in (red colors) and not involved (green colors) in RsfA-binding based on results from subfigure (C). (d) Residues of L14 highlighted that are involved in formation of intersubunit bridges with the 16S rRNA of the 30S subunit (bridge B5 (green colors), bridge B8 (red colors)) [15]. (B) A docking model of L14 on the *E. coli* 50S subunit with bound RsfA. Critical L14 residues that mediate RsfA interaction (or that contact 16S rRNA) are colored in red according to A(c) and A(d). When RsfA is bound to L14 on a 50S subunit, 30S subunit joining is sterically blocked, clearly visible in B(b) as shown by the structural overlap of RsfA (dark blue) and the 30S subunit. A model of the ribosome with bound RsfA is available as Dataset S1. (C) L14 interaction epitope mapping. Amino acids (see Figure 2A(c)) were mutated to alanine and the constructs tested by Y2H experiments. WT, wild type L14 construct; mutated residues and their positions are indicated. In the experiment, all bait constructs were simultaneously tested for reporter gene self-activation. No construct resulted in self-activation (data not shown). T97A, R98A, or K114A mutations (highlighted by arrows) abolished or weakened RsfA binding as indicated by 3-AT titrations; all other tested L14 mutation constructs are comparable to wild type L14.

In order to map the exact binding site of RsfA we used the LRS 3D structure (PDB id: 2AWB [14]: first, we identified amino acids of L14 that (i) are highly conserved (Figure 2A(a) and 2A(b)) and that (ii) are located on the surface exposed towards the 30S small subunit interface. These criteria identified T97, R98, K114, and S117. (Figure 2A(b,c)). In fact, docking a homology model of RsfA and a crystal structure of L14 predicted these residues to be at their interaction interface (Figure 2B). In order to test whether the identified residues of L14 are indeed essential for the L14-RsfA interaction, we substituted T97, R98, K114, and S117 with a single alanine each and tested these L14 constructs if they still bound RsfA by another Y2H experiment (Figure 2C): the K114A and T97A mutants lost the interaction with RsfA already in the presence of 0 to 1 mM 3-AT, while in R98A the interaction was lost at 10 mM and higher concentrations. S117A did not appear to affect the interaction. Several control mutations including moderately conserved amino acids (D80A, F100A, E121A) and none-conserved ones (R49A, K51A) did not show any difference in the Y2H assay compared to the assayed wild type L14 (Figure 2A, 2C).

In summary, the interaction epitope assay confirms that the docking model (Figure 2B) is largely correct. The RsfA-interaction epitope of L14 involves the highly conserved residues K114, T97, and R98 (but not S117) while K114 and T97 are the most critical ones. Notably, T97 and R98 are involved in bridge B8 (Figure 2A(d)) that contacts the small ribosomal subunit [15]. The docking model predicts that binding of RsfA to these residues, as a consequence, would sterically interfere with ribosome subunit joining (Figure 2B(b)) and thus might block translation.

### RsfA confers a strong selective advantage under natural growth conditions

**Figure 3:**
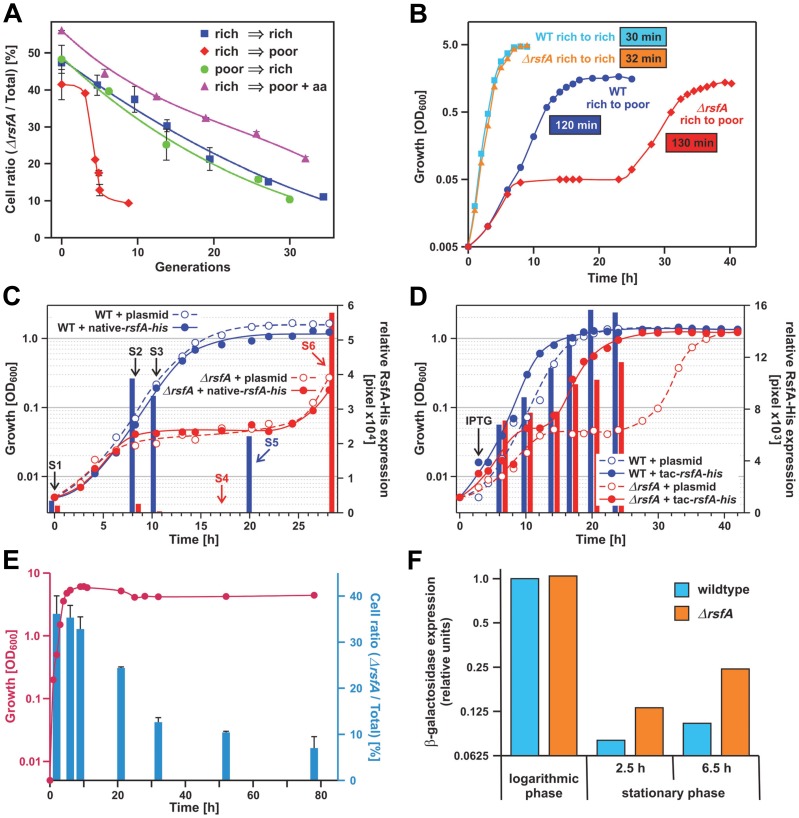
RsfA inhibits translation during both stationary phase and the transition from rich to poor media. (A) Growth competition experiment: equal numbers of *E. coli* wild type and *ΔrsfA* cells derived from an overnight LB-culture were mixed and grown in rich medium (LB, rich→rich), poor medium (M9, rich→poor) and poor medium plus 2% casamino acids as indicated (rich→poor+aa). Growth was maintained in log phase conditions by regular dilutions in the corresponding media. Shown is the fraction of viable *ΔrsfA* mutant cells in the total cell population. (B) Wild type and mutant strains were grown overnight in rich medium (LB) and then diluted in rich (rich→rich) or poor M9 medium (rich→poor). The generation time was derived from the slopes of the regression lines made of the points indicating the logarithmic phase. The errors of the generation-time determinations are below ±5%, *i.e.* generation times of 30 and 32 min are not significantly different. (C) Wild type and mutant strains transformed with a plasmid harboring the gene for RsfA fused with a His-tag under control of the native promoter or the corresponding empty plasmid were grown overnight in rich medium (LB) and then diluted in poor M9 medium. At certain times samples were withdrawn (S1–S6) and the relative amount of RsfA was quantified by Western-blot (represented with bars). S1–S3: samples were analyzed from both strains. S4–S6: samples were analyzed only from wild type (blue) or mutant strain (red). (D) Same as (C) but using a plasmid with a His-tagged RsfA gene under a tac promoter. After ∼3 h incubation in M9 medium 0.2 mM IPTG (final concentration) was added to all strains in order to induce expression from the tac promoter. (E) Viability competition similar to the growth competition described under (A) but in a batch culture without dilution. Red, growth of the mixture of *ΔrsfA* and WT strains; blue, the fraction (in %) of the mutant strain. (F) Expression of β-galactosidase as reporter to test translational activity of logarithmic and stationary phase cells in WT and *ΔrsfA* cells induced by 2% arabinose. Induction time was 3 h in logarithmic and 2.5 and 6.5 h in stationary phase. The expression level was derived from the band-intensity on a gel (Coomassie-stained SDS-PAGE).

Although RsfA is phylogenetically highly conserved, its gene deletion has been reported not to result in any obvious growth disadvantage in *E. coli* [7], [16]. We designed a sensitive growth experiment, which compares the WT and the *rsfA* deletion strain under competitive growth conditions: we mixed equal amounts of both cell types and monitored the populations at constant time intervals under log-phase conditions. Figure 3A demonstrates that the amounts of mutant cells decreased continuously. In other words, WT cells in rich medium steadily overgrew the mutant cells leaving only about 10 to 25% of mutant cells after 35 generations. This modest effect reveals that RsfA mutant cells suffer from a disadvantage when competing with WT cells. Strikingly, a much stronger difference was observed, when cells grown in rich medium were diluted in minimal medium: the WT strain overgrew the mutant *ΔrsfA* strain within only five generations. The opposite growth transition (poor→rich media) is better tolerated by the mutant strain. The addition of amino acids to the minimal medium completely rescues this striking growth defect of the *rsfA* mutant in the rich→poor media transition (see Discussion).

These strong defects seen with the *ΔrsfA* strain in minimal medium rather than in rich medium should be evident also in a direct determination of the doubling times of wild type *versus* mutant in separate cultures. In rich medium the generation times of WT and mutant strains were not significantly different (30 and 32 min, respectively; Figure 3B). However, a change from rich to poor medium revealed a dramatic difference: initially the *ΔrsfA* mutant strain showed a growth like the WT strain for about 7 h, but then growth was abrogated for about 14 h before it resumes almost with the same doubling time as the WT strain (130 *versus* 120 min). The growth block for many hours demonstrates that the lack of the *rsfA* gene poses a serious adaptation problem on the cells after a transition from rich to poor medium.

It has been reported that the *rsfA* (formerly *ybeB*) knock-out can cause a defect in cell separation in a distinct genetic background, and this defect can be complemented with genes of the *rsfA* operon downstream of the *rsfA* gene indicating a polarity effect of the *rsfA* deletion [17]. Therefore, we tested whether we can complement the strong mutant phenotype observed in Figure 3A and 3B by introducing a plasmid carrying the *rsfA* gene. If so, it would prove that the mutant phenotype is caused by the absence of the RsfA factor. To this end, we removed the kanamycin cassette in place of the chromosomal *rsfA* gene and introduced a plasmid with the *rsfA* gene under the native promoter; the expressed RsfA carried a His-tag at the C-terminus to monitor the expression by anti-His antibodies. Figure 3C demonstrates that the mutant phenotype could not be cured probably due to the fact that after the shift to the poor medium RsfA was not sufficiently expressed, whereas taking up growth after 30 h was accompanied by a strong RsfA expression (see red bars in Figure 3C). Therefore, we performed the same experiment but now with the *rsfA* gene under a tac promoter. The forced RsfA expression could heal the mutant phenotype (Figure 3D; red closed circles). We conclude that (i) the RsfA expression is regulated in a way we do not yet understand, and (ii) that the lack of RsfA is responsible for the mutant phenotype.

Figure 3A and 3B demonstrate that mutant and WT strains showed almost the same growth behavior under log-phase conditions in rich medium (LB). But what happens in a batch culture, when a mixture of both strains reaches the stationary phase in rich medium and protein synthesis has to be down regulated? This was tested in the next experiment. The stationary phase is reached after about 7 h (red line in Figure 3E). At various time points aliquots were taken and the fraction of *ΔrsfA* mutant strains were determined (blue bars). Until reaching the stationary phase the fraction of mutant cells remains constant at about 35%, but thereafter the fraction of mutant cells sharply declined to less than 10%. This viability competition assay indicates that the mutant cells have serious problems to form stable stationary-phase cells.

The experiments shown in Figure 3A–3E disclose two strong phenotypes caused by the lack of RsfA: (i) The cells adapt poorly after the transition from rich to poor media, and (ii) the viability of cells is dramatically impaired during the stationary phase, eventually causing cell death.

### RsfA acts as a negative modulator of protein translation *in vivo*

Given RsfA's physical association with the large ribosomal subunit/L14, we wondered whether RsfA has an effect on protein synthesis. To this end we expressed β-galactosidase (as an L-arabinose inducible reporter) in an *E. coli* gene deletion strain (*ΔrsfA*) and wild type (WT) cells. At stationary phase the β-galactosidase expression was strongly repressed in wild type cells as expected (Figure 3F). In striking contrast, the *ΔrsfA* mutant exhibited a significant accumulation of β-galactosidase in the stationary phase. These results demonstrate that RsfA acts as a negative modulator of protein translation *in vivo* in the stationary phase. Together with the viability assay (Figure 3E) these results suggest that silencing protein synthesis plays an important role for reorganization of the metabolic conversion on the way to the stationary phase.

### RsfA is a ribosomal silencing factor that interferes with the association of ribosomal subunits

**Figure 4:**
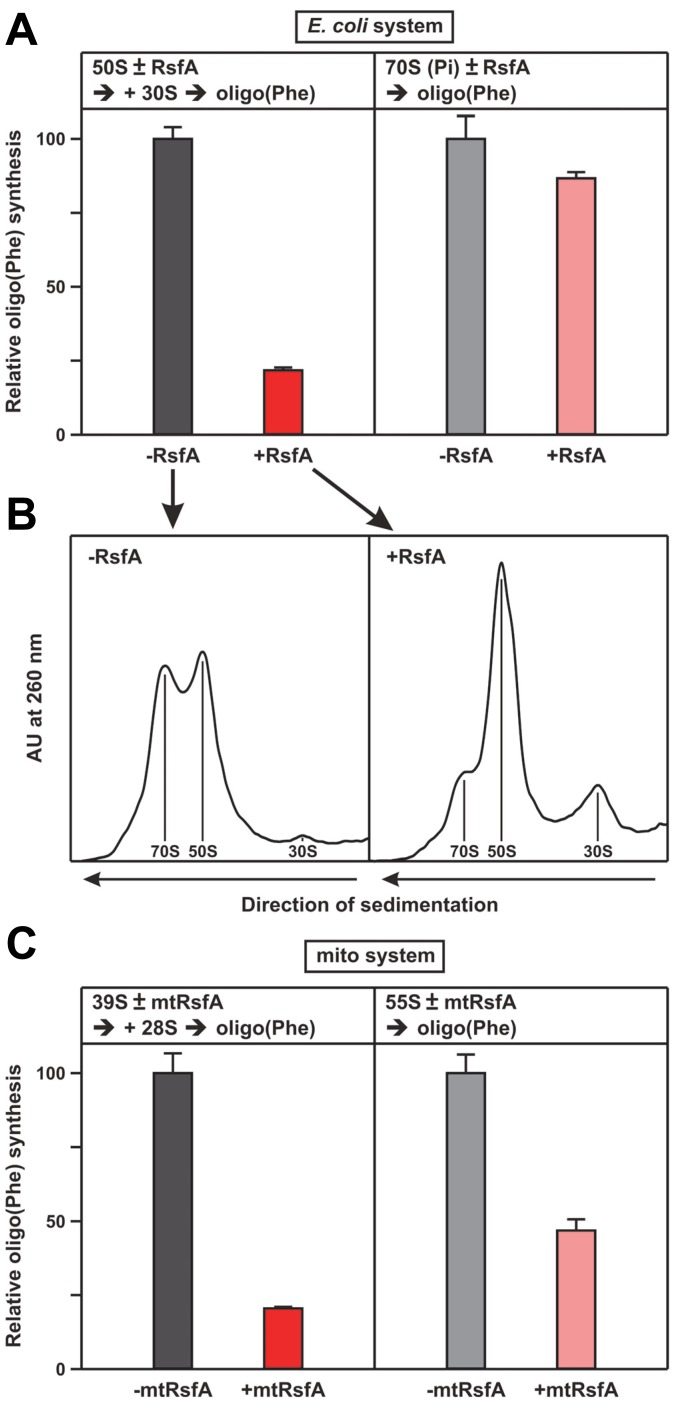
RsfA inhibits translation by blocking ribosomal subunit joining. (A) Oligo(Phe) synthesis in a pure system containing pre-charged Phe-tRNAs (ten times over ribosomes), 30S and 50S subunits and the purified factors EF-Tu, EF-Ts and EF-G plus/minus RsfA from *E. coli*, 100% corresponds to 7 Phe incorporated per ribosome. Left panel, when indicated RsfA was added to the 50S subunits, before 30S subunits were added starting oligo(Phe) synthesis. Right panel, AcPhe-tRNA was bound to 70S ribosomes in the presence of poly(U) before the addition of RsfA. (B) Sister-aliquots from the same samples shown in (A) were analyzed on a sucrose gradient before oligo(Phe) synthesis. The presence of RsfA significantly reduces the fraction of 70S ribosomes. (C) Oligo(Phe)-synthesis as in (A) but with purified mitochondrial components (pig liver) and human mtRsfA (C7orf30). 39S and 28S indicate the large and small ribosomal subunits, 55S the associated mitochondrial ribosomes. For details see Experimental Procedures.

Next we tested whether RsfA interferes with ribosomal elongation *in vitro* using a highly resolved *E. coli* system just containing purified elongation factors EF-Tu, EF-Ts, EF-G, purified precharged [^14^C]Phe-tRNA, poly(U) programmed ribosomes and GTP as energy source. We added 30S subunits to an excess of 50S subunits in order to facilitate association to 70S ribosomes. Purified RsfA suppressed the translational activity dramatically down to about 20%, when RsfA was added to the 50S subunits before the oligo(Phe) synthesis (Figure 4A, left panel). To test whether RsfA blocks ribosomal activities *via* interfering with association of the subunits as suggested by our protein docking model (Figure 2B), we subjected an aliquot to a sucrose-gradient analysis before incubating for oligo(Phe) synthesis (Figure 4B). The gradients demonstrate that in the absence of RsfA clearly more 70S ribosomes are formed on the cost of ribosomal subunits. However, when RsfA was added to programed 70S ribosomes carrying an AcPhe-tRNA at the ribosomal P site, no inhibition was observed indicating that RsfA does not interfere with ribosomal functions during the elongation phase (Figure 4A, right panel). We conclude that RsfA blocks association of the ribosomal subunits to functional 70S ribosomes.

Corresponding experiments with the translational elements of mitochondrial ribosomes from mammalian cells (pig liver) confirmed these results. In the presence of purified mitochondrial factors mtEF-Tu, mtEF-Ts, mtEF-G1, poly(U) and [^14^C]Phe-tRNA oligo(Phe) synthesis was severely reduced upon addition of the mitochondrial RsfA orthologue C7orf30 (mtRsfA; Figure 4C). The results suggest that the function of RsfA is conserved from bacteria to eukaryotic mitochondria.

## Discussion

The cellular synthesis machinery runs at high speed in the exponential (logarithmic) phase of bacterial growth. The growth rate slows in semi-log phase and finally comes to a halt at higher cell density in the stationary phase, usually caused by nutrient depletion. Several bacterial factors bind to ribosomes and thus support the dormant state of the ribosomes in the stationary phase, such as the ribosome modulation factor (RMF), hibernation promoting factor (HPF) or stationary-phase-induced ribosome-associated protein (SRA) [18], [19], [20], [21]. RMF (homologues exist only in the γ-proteobacteria) alone or together with the more broadly distributed HPF are essential for the formation of 70S dimers in the stationary phase, so called 100S particles; an inactivation of the RMF gene causes a viability defect at prolonged periods in stationary phase [22], [23]. Phenotypical effects of knock-out strains concerning the other factors have not been reported.

A first analysis of RsfA-binding partners identified a group of proteins including a number of ribosomal proteins [6]. Similarly, other groups suggested various ribosomal proteins as binding partners [3], [4], [5], the common denominator being that all proteins were derived from the large subunit. Thorough analyses presented here identified the ribosomal protein L14 as the docking station (Figure 1B–1G, Figure 2), and mutation of conserved amino acid residues of L14 at the surface of this protein abolished RsfA binding, clearly demonstrating L14 as the binding protein (Figure 2). Interestingly, the three most conserved residues of RsfA as shown by the multiple sequence alignment (Figure S1A) are located at the interface with L14 predicted by docking. The three residues are W120, D124 and R140 (alignment numbers), corresponding to residue numbers W77, D81 and R95 in *E. coli* RsfA. D81 is predicted to be in direct contact with R98 of L14 that was shown to disrupt the interaction when mutated. Another such critical residue, K114 of L14, is predicted to be in contact with a fairly conserved residue with RsfA L103 (position 148 in the alignment).

The only other known protein that like RsfA also docks to the ribosomal protein L14 of eukaryotic ribosomes is the so-called initiation factor eIF6, which is not a homologue to RsfA and is thought to block ribosome association in archaea and in eukaryotes from yeast to man [24], [25], [26], [27], [28], [29]. However, in eukaryotes eIF6 is rather a 60S assembly factor and plays an essential role in the late pre-25S rRNA processing and the export of the 60S subunit from the nucleolus to the cytoplasm [30]. Depletion of eIF6 is eventually lethal, in contrast to RsfA. Interestingly, eIF6 is restricted to the eukaryotic nucleus/cytoplasm and to archaea [27], while RsfA is present in almost all bacteria and their descendent eukaryotic organelles (Figure 1A).

Studies with the human mitochondrial homologue of RsfA, C7orf30, have recently suggested that this protein is involved in ribosomal assembly and/or translation [5], [8]. Our results do not indicate any assembly defects as deletion strains of *rsfA* appear to have perfectly assembled ribosomes (sucrose gradients not shown) and actually translate as well as wild type strains at logarithmic phase (Figure 3F). In addition, we could show that C7orf30 inhibits translation by mitochondrial ribosomes (Figure 4C). It remains possible that C7orf30 has multiple roles in mitochondria or that its role in ribosome assembly is indirect.

In rich medium bacterial cells produce proteins at maximum rates to sustain cell division. Furthermore, bacterial cells take up many metabolic precursors such as amino acids and thus block corresponding synthesis pathways. In contrast, in poor/minimal medium protein synthesis must be down-regulated in a concerted fashion in order to save energy and resources, and at the same time many synthesis pathways such as those for the synthesis of amino acids have to be switched on [31], [32]. The results presented here suggest that RsfA plays a prominent role in this down-regulation by silencing ribosome activities. We observe two strong phenotypes with the *ΔrsfA* strain: (i) the viability is strongly impaired in the stationary phase (Figure 3E) and (ii) after a transition from rich to poor media the adaptation phase lasts more than 10 hours before resuming growth again in striking contrast to WT cells (Figure 3B), which overgrow the mutant strain in a few generations. Just adding casamino acids to the minimal medium relieves the strong growth defects of the *ΔrsfA* strain (Figure 3A). Adding amino acids will switch off most of the amino-acid synthesis pathways similar to the situation during the logarithmic phase in the presence of rich medium, when the silencing effect of RsfA is not strictly required. In contrast, during starvation and in the absence of ribosomal silencing (*ΔrsfA*), energy would be wasted affecting the conversion of the metabolic network, eventually causing deleterious growth defects. Accordingly, protein synthesis is seriously attenuated in the stationary phase, when RsfA is present (i.e. wild type cells) in contrast to protein synthesis in the *ΔrsfA* strain (Figure 3F). Attenuation of protein synthesis by RsfA seems to be of utmost importance for reorganization the metabolic state on the way to the stationary phase, since the absence of this factor threatens seriously the viability in the stationary phase (Figure 3E), and it explains the well-known effect that ribosomes are much less active, when derived from the stationary rather than from log-phase cells [33].

When RsfA is added to ribosomal subunits it blocks 70S formation and thus protein synthesis (Figure 4A and 4B), whereas the factor does not interfere with the elongation phase of protein synthesis when added to ribosomes that have passed the initiation phase (Figure 4A, right panel). We conclude that RsfA, as a ribosomal silencing factor, is damping the translational activity under restricted energy (stationary phase) or nutrient conditions (growth in poor medium) thus harmonizing translation with the general metabolic state, *i.e.* RsfA works in line with the stringent response [34] and thus plays a key role in the physiology of the stationary phase and the translational adaptation during the transition from rich to poor medium.

**Figure 5:**
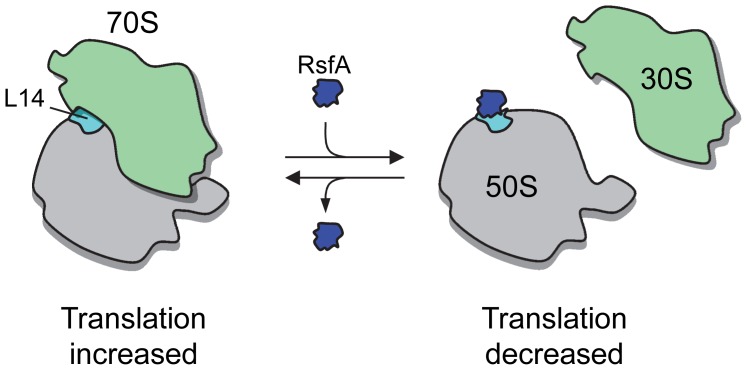
A model of RsfA action. In rich medium and during exponential growth, RsfA is either not present or not active, so that protein synthesis is fully active. In starving cells, RsfA binds to ribosomal L14 and, as a consequence, blocks ribosomal subunit joining and thus protein synthesis.

Our experiments suggest a direct silencing effect of RsfA sketched in Figure 5: when the ribosomal activity should be silenced, RsfA binds to the ribosomal protein L14 at the interface of the large subunit and by impairing association of the ribosomal subunits translation is hampered. We demonstrated that RsfA damps the ribosomal elongation in bacterial and mammalian mitochondrial systems (Figure 4A and 4C). The importance of RsfA in eukaryotic organelles is indicated by the fact that a mutation in the gene of the RsfA orthologue Iojap in *Zea mays* leads to irregular albino patterns on maize leafs and germless seeds due to failure of proplastids to differentiate into chloroplasts [35], [36], [37], [38]. Photosynthesis and respiration can vary enormously in plastids and mitochondria, respectively, and as suggested by the experiment shown in Figure 4C, the RsfA orthologue might accordingly regulate protein synthesis in these organelles using the mechanism suggested here.

## Materials and Methods

### Cloning

ORFs were cloned into pDONR207 by using the Gateway Technology (Invitrogen). *Zea mays* cDNA was kindly provided by F. Hochholdinger (Tübingen, Germany), HeLa cDNA by O. Kassel (Karlsruhe, Germany), *S. pneumoniae TIGR4* DNA by D. Nelson (UMBI, MD, USA), *T. pallidum* DNA by T. Palzkill (Houston, USA), and *Synechocystis PCC 6803* DNA by T. Lamparter (Karlsruhe, Germany). All ORFs were cloned with a stop codon at the 3′-ends. Entry plasmids were sequenced, shuttled into expression vectors (see below), and finally verified by PCR reactions. For the interologous tests *E. coli* ORFs were kindly provided as pENTR/Zeo clones by S.V. Rajagopala [39] except for RsfA and L14 which have been cloned in this study.

*E. coli* L14 (b3310) alanine substitutions were directionally introduced by performing standard fusion PCR reactions using mutagenic primers. For cloning PrimeStar HS DNA Polymerase was used (Takara Bio Inc.).

### Yeast two-hybrid assays

Entry plasmids were recombined with the bait and prey vector pGBKT7g and pGADT7g (Clontech) [40]. These were individually transformed into the haploid yeast strains AH109 and Y187 [41], [42]. After mating the haploids and enrichment of diploids, yeast growth was observed on solid starvation medium lacking Leucine, Tryptophan, and Histidine. The medium contained various concentrations of 3-AT (0 to 100 mM). Detailed procedures were done as described elsewhere [43].

In case of the L14-interaction epitope mapping experiment bait and prey plasmids were sequentially cotransformed into haploid yeast strain CG-1945 (Clontech) and then assayed as described above.

### Pull down assays

ORFs were shuttled from entry plasmids into pNusA (Santhera, Liestal, Switzerland), pETG-40A, or pETG-30A (EMBL, Heidelberg, Germany) and transformed or co-transformed into *E. coli* BL21(DE3) (combinations, see main text, Figure 1D and 1E and Figure S3A). Proteins were expressed following standard protocols. Cell pellets were lysed in 500 µl buffer (50 mM Tris-HCL pH 8.0, 100 mM NaCl, 50 µg/ml chicken egg white lysozyme, 50 µM PMSF, Sarcosyl/Triton-X 100 0.1%, each) and then sonicated and centrifuged. The supernatants were used for pull-down experiments: for *E. coli* RsfA and L14 corresponding volumes of 50 µg soluble protein fractions of co-expressed proteins were applied to beads and aliquots saved as input controls. For human and *Zea mays* proteins 25 µg soluble fractions were mixed and then applied to the beads. MBP fusions were co-purified with their GST baits on 20 µl glutathione beads and NusA-tagged preys with their MBP fusions on 20 µl amylose beads under buffer conditions indicated above but w/o lysozyme. Binding occurred at room temperature for 30 min. Then, the beads were washed and finally boiled in 50 µl Laemmli buffer. 10 µl of output (∧ = 10 µg protein input) and 10 µg input samples were separated by SDS PAGE using 12% gels. Proteins were transferred onto a polyvinylidene fluoride membrane by semi-dry Western blotting. The recombinant bait and prey proteins were labeled by standard immunodetection procedure and then analyzed by enhanced chemiluminescence.

### *In vivo* localization and BiFC assays

Human C7orf30 (mtRsfA) and L14_mt_ full-length ORFs were cloned into pcDNA3.1-HA-mCherry [44], pcDNA3.1(+)-HA-VN, and pcDNA3.1(+)-HA-VC [45] (Note: an N-terminal HA tag from the vector backbones was removed under consideration that the native mitochondrial localization peptides of mtRsfA ( = C7orf30) and L14_mt_ are N-terminally exposed).

For localization studies, Hela cells were transfected (100 ng, each plasmid) with mCherry-tagged C7orf30 or L14_mt_ using Promofectin (Promokine, Germany). 100 ng pECFP-Mem (Clontech) was co-transfected to stain cell membranes. 24 h later, MitoTracker Green FM (100 nM f.c., Invitrogen) was added. After washing, DRAQ5 (1∶2,000, Biostatus) was added fur nuclear staining.

For BiFC assays [46], Hela cells were prepared correspondingly. Exceptions: Mitotracker staining was not done and instead of localization constructs, cells were co-transfected with BiFC plasmid constructs (50 ng, each) in combinations as given in Figure 1G.

30 min post DRAQ5 administration cells were analyzed by fluorescence microscopy using a Zeiss LSM 510 Meta confocal laser scanning microscope.

### Conservation of L14 residues

Multiple alignments were generated using ClustalW [47] with the L14 amino acid sequences from *E. coli*, *T. pallidum*, *S. pneumoniae*, *Synechocystis PCC 6803*, *C. jejuni*, *H. sapiens*, *Zea mays*, *Chromobacterium violaceum, Bacillus halodurans*, and *S. cerevisiae* using default parameters. Based on that alignment the conservation scores were calculated with the ConSurf Server [48]. 3D images (Figure 2A) were presented using PyMol 1.5 (http://pymol.org).

### Protein docking

Structures of unbound proteins: the *E. coli* L14 structure was taken from 2AWB PDB entry, chain K [14]. Because the crystal structure of *E.coli* RsfA is not available, we used I-TASSER server [49] to build a model of that protein. The server built a single model using as templates 2ID1_A and 2O5A_A. The server has estimated the accuracy of the model as 0.90±0.06 (TM-score) and 1.6±1.4 Å (RMSD).

An unconstrained rigid body docking was performed of individual L14 and RsfA structures with GRAMM-X [50]. We then used the coordinates of L14 to superimpose 100 top scored docking models onto the entire 70S unit (2AWB and 2AW7 PDB IDs). Then, each model was evaluated for the backbone clashes between the predicted RsfA position and the rest of the 50S subunit. We defined a clash as having less than 2 Å distance between backbone atoms in order to tolerate some degree of unknown conformational re-arrangement of the 50S components that were not used in docking. Model #17 was the first one in order of the docking score where RsfA had no clashes with other parts of 50S (parts not seen by the docking procedure). Model #17 contained certain surface exposed amino acid residues of L14 that are highly conserved (Figure 2B). To test whether these are involved in mediating the interaction with RsfA they were subjected to alanine substitution constructs (see above and Figure 2A) and analyzed in Y2H experiments (Figure 2C). The interface contacts were defined as having less than 4.6 Å distance between any heavy atoms of the docking subunits. We used PyMol 1.5 (http://pymol.org) for the post-docking analysis and graphics.

### β-galactosidase expression in logarithmic and stationary phase

*ΔrsfA* (b0637) [16] and wild type (BW25113) were transformed with a β-galactosidase reporter plasmid, pBAD24-lacZ-HA (based on pBAD24HA) [51], [52] and selected on LB agar containing 50 µg/ml ampicillin. Both were grown overnight in LB in the presence of 50 µg/ml ampicillin and 0.4% glucose as inhibitor of leaky expression. For stationary phase expression cultures were centrifuged at 5,000 rpm (15 min) and pellets were resuspended in the cell-free supernatant of an LB overnight culture (BW25113/*ΔrsfA*, no plasmid) lacking glucose. β-galactosidase expression was induced with 2% arabinose; the resuspension was adjusted to the same cell density as the previous stationary-phase culture. For logarithmic phase expression overnight cultures were centrifuged at 5,000 rpm for 15 min and pellets were resuspended in fresh LB medium (no glucose) with 50 µg/ml ampicillin for both strains. Cultures were then diluted to OD_600_ = 0.05 and grown for 2 h. β-galactosidase expression was induced by adding 2% arabinose to the medium. The cultures were shaken at 37°C. Every hour 300 µl suspension was withdrawn, 100 µl from it was loaded into a well of a 96-well plate (flat bottom) and the growth was followed by monitoring the extinction at 600 nm (ELISA spectrophotometer). The rest of aliquots were centrifuged at 12,000 rpm for 5 min and pellets were resuspended in 20 µl loading buffer (2×) Tris-glycine SDS and incubated at 95°C for 5 min to denature proteins. Samples were loaded on SDS-polyacrylamide gel (10%) and the β-galactosidase amount was quantified as relative protein-band intensity using ImageJ 1.45.

### Growth/viability competition assay

For growth competition assays (Figure 3A) the same amount of cells from overnight cultures of wild type and *ΔrsfA* strains were mixed, yielding a final OD_600_ of 0.01 in a volume of 5 ml, and incubated with mild shaking either in LB (rich) or M9 medium with 0.4% glucose (poor). Aliquots were withdrawn every 3 h or 6 h or 24 h (depending on the growth rate) and OD_600_ was measured. Simultaneously, dilutions to approximately 5,000 cells/ml (according to the assumption that 1 OD_600_ corresponds roughly to 10^9^ cells) were made and 100 µl of each was plated in duplicates on either LB plates or LB plates containing 25 µg/ml kanamycin. The number of colonies (*ΔrsfA* contained a kan^R^-cassette, WT not) was counted after incubation at 37°C for overnight. For viability competition experiment in stationary phase (LB medium; Figure 3F) *ΔrsfA* mutant and wild type strain were separately grown overnight. Subsequently two cultures were diluted to OD_600_ = 0.005 and incubated with shaking till 0.5 OD_600_. Then two cultures were mixed and the fitness of *ΔrsfA* was monitored as numbers of colonies on LB plates (mutant and wild type colonies) and LB plates containing kanamycin (only mutant colonies) after 2, 6, 9, 21, 32, 52, 78 hours of incubation at 37°C.

### Romoval of the kan^R^-cassette in the *Δ*rsfA strain

The kanamycin resistance gene that substituted the *rsfA* was removed by introducing a flippase-encoding plasmid pCP20 as described elsewhere [53]. The successful flip-out was verified by a genotyping PCR.

### Media shift rich to poor

For the media shift (Figure 3B) wild type and *ΔrsfA* strains were grown overnight in LB medium (rich) and then diluted in either LB (rich) or M9 medium (poor) yielding a start OD_600_ = 0.005. Cultures were incubated at 37°C with shaking (200 rpm) and growth was monitored measuring the OD_600_ over a time of up to 40 hours.

For curing the phenotype of the *ΔrsfA* strain during the transition from rich to poor (Figure 3C and 3D) *ΔrsfA* cells lacking the kanamycin resistance gene and wild type cells were transformed with a plasmid harbouring the gene coding for RsfA fused with a C-terminal His-tag under control of either the native promoter or the IPTG inducible tac-promoter and with the corresponding empty plasmid.

The transformed strains were grown overnight in rich (LB) medium at 37°C and then diluted in poor M9 medium yielding a start OD_600_ = 0.005 and incubated like described above. At several time points samples were withdrawn and the expression of RsfA was analysed after SDS-PAGE and Western-blot using an antibody directed against the His-tag. The intensity of the RsfA-His bands was quantified using ImageQuant 5.2 and normalized for correction of the input to a non-altered protein band of the Coomassie stained gel.

### Expression and purification of *E. coli* RsfA and human mtRsfA

The gene coding for *E. coli* RsfA (b0637) was expressed as an N-terminal His_6_ tag fusion in *E. coli* BL21(DE3). Expression was induced at OD_600_ = 0.4 with 0.1 mM IPTG and carried out for 2 h at 30°C to decrease the formation of inclusion bodies. The soluble protein was purified via nickel-nitrilotriacetic-acid-agarose (Qiagen, according to the manufacturer's manual) and anion exchange chromatography (Source 15Q, GE Healthcare). The purified protein was dialyzed against 20 mM Hepes, 6 mM Mg-acetate, 150 mM K-acetate, 4 mM β-mercaptoethanol, pH 7.6 at 0°C.

The gene coding for the mature human mitochondrial RsfA (C7orf30; amino acids 23–234) was expressed and the protein purified like the *E. coli* RsfA orthologue.

Both proteins were expressed using the Gateway System-compatible plasmid pHGWA [54].

### Isolation of ribosomal components

Ribosomes and ribosomal subunits were prepared from *E. coli* strains CAN20-12E [55] as described [56]. Preparation of mammalian mitochondrial ribosomes and ribosomal subunits (pig liver) followed [57] with minor modifications. Hepes-buffer and TCEP were utilized instead of Tris-buffer and 2-mercaptoethanol, respectively. Isolation of mitochondrial factors are described in [58].

### Poly(U)-dependent oligo(Phe) synthesis with precharged Phe-tRNA and sucrose gradient analysis

18 pmol 50S ribosomes were incubated with 180 µg poly(U) with or without 360 pmol RsfA in 90 µl for 10 min at 37°C in binding buffer (20 mM Hepes, pH 7.6 at 0° C, 4.5 mM Mg-acetate, 150 mM K-acetate, 4 mM β-mercaptoethanol, 2 mM spermidine, 0.05 mM spermine, H_20_M_4.5_K_150_SH_4_Spd_2_Spm_0.05_). Reaction was further incubated with 10 pmol 30S ribosomes for 10 min at 37°C and then analyzed in poly(U) dependent oligo(Phe) synthesis and sucrose gradient centrifugation.

15 µl of the reaction was used for oligo(Phe) synthesis. 2.4 pmol EF-G together with the ternary complex mix were added yielding 30 µl in binding buffer H_20_M_4.5_K_150_SH_4_Spd_2_Spm_0.05_. The ternary complex mix contained in 15 µl 30 pmol [^14^C]Phe-tRNA^Phe^, 45 pmol EF-Tu, 45 pmol EF-Ts, 3 mM GTP and was preincubated 5 min at 37°C. Incubation was at 30°C for 2 min and 12.5 µl aliquots were precipitated with TCA, incubated at 90°C in the presence of 2 drops of 1% (w/v) BSA and filtered through glass filters and counted.

60 µl of the reaction was mixed with 40 µl H_20_M_4.5_K_150_SH_4_Spd_2_Spm_0.05_ and loaded onto a 10–30% sucrose gradient prepared in the same buffer. Centrifugation was carried out at 42,000 rpm for 4 h in an SW60 rotor. The gradient was pumped out from bottom to top and the A_260_ was measured to obtain the ribosome profile.

The corresponding assay with mitochondrial components from pig liver was performed in H_20_M_4.5_K_150_SH_4_Spd_2_Sp_0.05_ pH7.5 (at 0°C). mtRsfA was pre-incubated with 2.5 pmol large subunit 39S in 80 molar excess over ribosomes, before the same amount of 28S subunits were added; likewise 2.5 pmol 55S ribosomes were incubated with the same amount of RsfA. EF-G1 was added in a 0.8-fold excess over ribosomes. 37.5 pmol of [^14^C]Phe-tRNA were present and the mitochondrial factors mtEF-Tu and mtEF-Ts, were added both in an excess of 1.5 over Phe-tRNA. The total volume was 100 µl, the main incubation 20 min at 30°C. The following processing was as described above.

The oligo(Phe) synthesis with reassociated 70S ribosomes (Figure 4A, right panel) was performed in the following way: 3 pmol 70 S ribosomes were incubated with 30 µg poly(U) and 6 pmol Ac-Phe-tRNA for 10 min at 37°C. When indicated 60 pmol RsfA was added and the oligo(Phe) synthesis performed as described above. The total volume was 20 µl, the mixture was incubated for 5 min at 37°C.

## Supporting Information

Dataset S1 PDB file of the ribosome with bound RsfA.
(ZIP)

Figure S1 Multiple sequence alignments of selected RsfA and L14 orthologues. (A) Protein sequences of RsfA orthologues which were shown to interact with L14, as well as orthologues from yeast and two species with available 3D-structures (*Chromobacterium violaceum*, PDB id: 2ID1 and *Bacillus halodurans*, PDB id: 2O5A). (B) Multiple sequence alignment of corresponding L14 protein sequences (only plastidal or mitochondrial L14 are shown for *Zea mays*, human and yeast, respectively). Amino acid residues of *E. coli* L14 that have been exchanged to alanine for interaction epitope mapping (Figure 2B) are highlighted in red (residue is involved in RsfA binding) and green (not involved in RsfA binding). Numbers on the left and right of the alignment sequences indicate the alignment start and stop positions, respectively. Consensus sequences shown at the top of each alignment were constructed with WebLogo V2.8.2 using default settings [60]. Multiple alignments were made using ClustalW2 [47]. Abbreviations: SYN (*Synechocystis sp. PCC 6803*), ZMA (*Zea mays*), TPA (*Treponema pallidum*), ECO (*Escherichia coli*), CVI (*Chromobacterium violaceum*), CJE, (*Campylobacter jejuni*), SPN (*Streptococcus pneumoniae*), BHA (*Bacillus halodurans*), SCE, (*Saccharomyces cerevisiae*), HSA (*Homo sapiens*).
(PDF)

Figure S2 Interologue tests. Pairwise Y2H interaction assays carried out with homologous protein pairs of *E. coli* that have been detected for RsfA in other studies (tested interactions and reference sets, see Table S1). Protein pairs were tested reciprocally (i.e., RsfA tested as bait and prey fusion) as quadruplicates on various concentrations of 3-AT. Baits are shown on top, preys are below in the legends. “C”, negative control: bait constructs are tested against the prey vector that does not contain any insert to check for reporter gene self-activation of the bait. Only the *E. coli* interaction of RsfA with L14 turned out to be conserved.
(PDF)

Figure S3 Interaction of RsfA-L14 in *E. coli, S. pneumoniae, and Synechocystis*. (A) Verification of *E. coli* RsfA-L14 interaction by a pull down assay. RsfA was tagged with glutathione S-transferase “G” and L14 with maltose binding protein “M”; i = input and o = output samples. (B, C) RsfA and L14 of *Streptococcus pneumoniae* TIGR4 (B) and *Synechocystis PCC 6803* (C) interact in Y2H assays. Protein pairs were tested in quadruplicates on various concentrations of 3-AT. C, control (empty prey vector).
(PDF)

Table S1 RsfA and L14 and their interaction are conserved in bacteria and eukaryotic organelles. (A) Known interactions of RsfA with L14 orthologues and physical association with the LRS. The table summarizes all known binary interactions among RsfA-L14 orthologous pairs as well as co-purified ribosomal protein complexes from this and other studies. RsfA-L14 interactions identified by binary methods are highlighted in light grey. RsfA orthologues co-purified with protein complexes/the ribosome are highlighted in dark grey. Abbreviations used: LRS (large ribosomal subunit), Y2H (yeast-2-hybrid), MS (mass spectrometry), Co-IP (co-immunoprecipitation), BiFC (bimolecular fluorescence complementation).
(DOC)

Table S2 RsfA interactions tested negatively with *E. coli* homologous protein pairs in a Y2H experiment (see Supplementary Figure S2). Orthologues from *T. pallidum* and *C. jejuni* were selected by MBGD orthologous protein groups [61]. The reference set gives the source of RsfA orthologous interactions they were primarily described in. Note, the interaction partners identified by Butland et al. are proteins that have been co-purified as protein complex of *E. coli* RsfA.
(DOC)
