# Supplementary figures and images for: RsfA (YbeB) Proteins Are Conserved Ribosomal Silencing Factors
Source: PLoS Genet. 2012 Jul 19;8(7):e1002815. doi: 10.1371/journal.pgen.1002815 (PMC3400551; doi:10.1371/journal.pgen.1002815)

Fig. S1

A

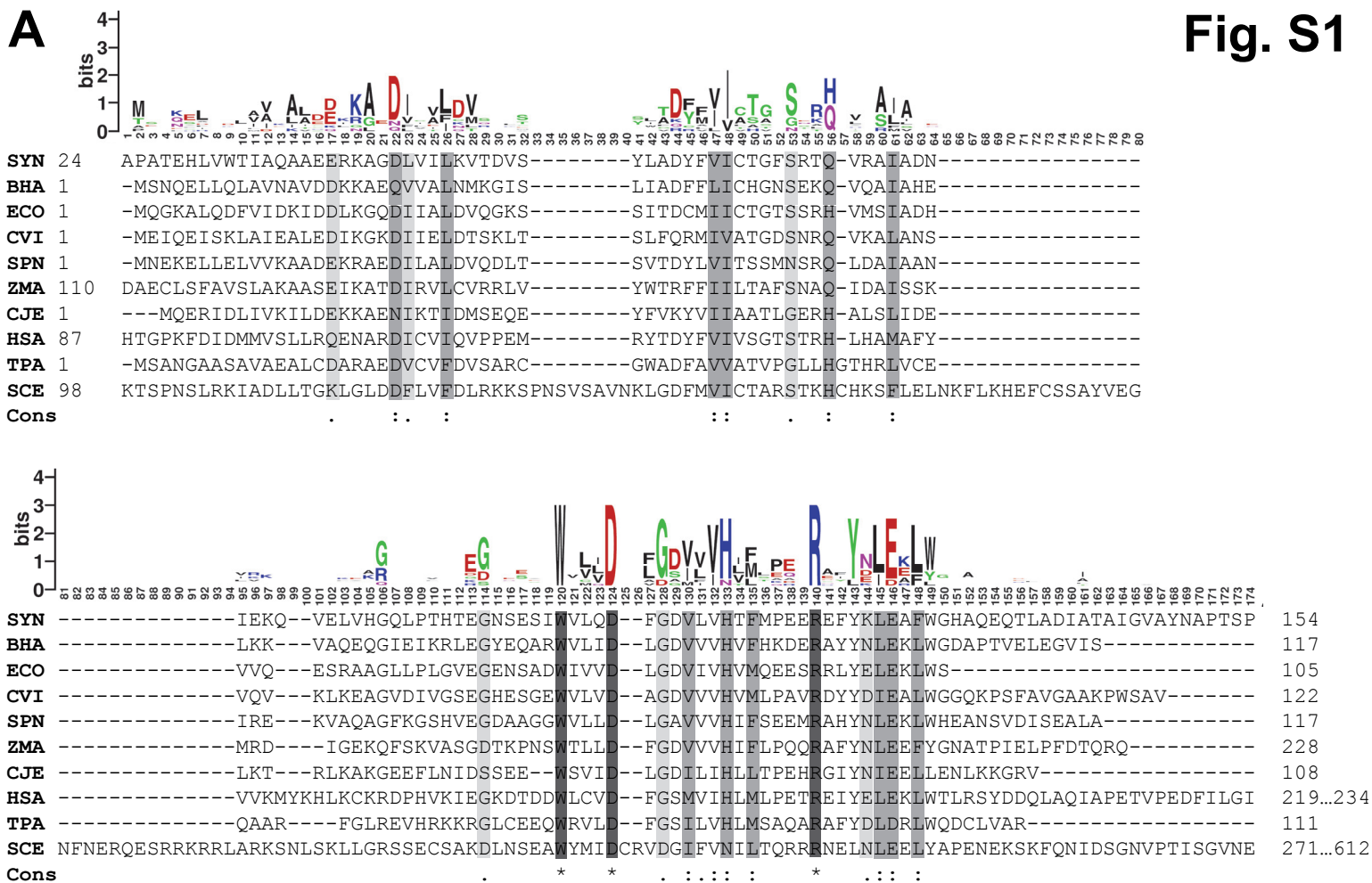

B

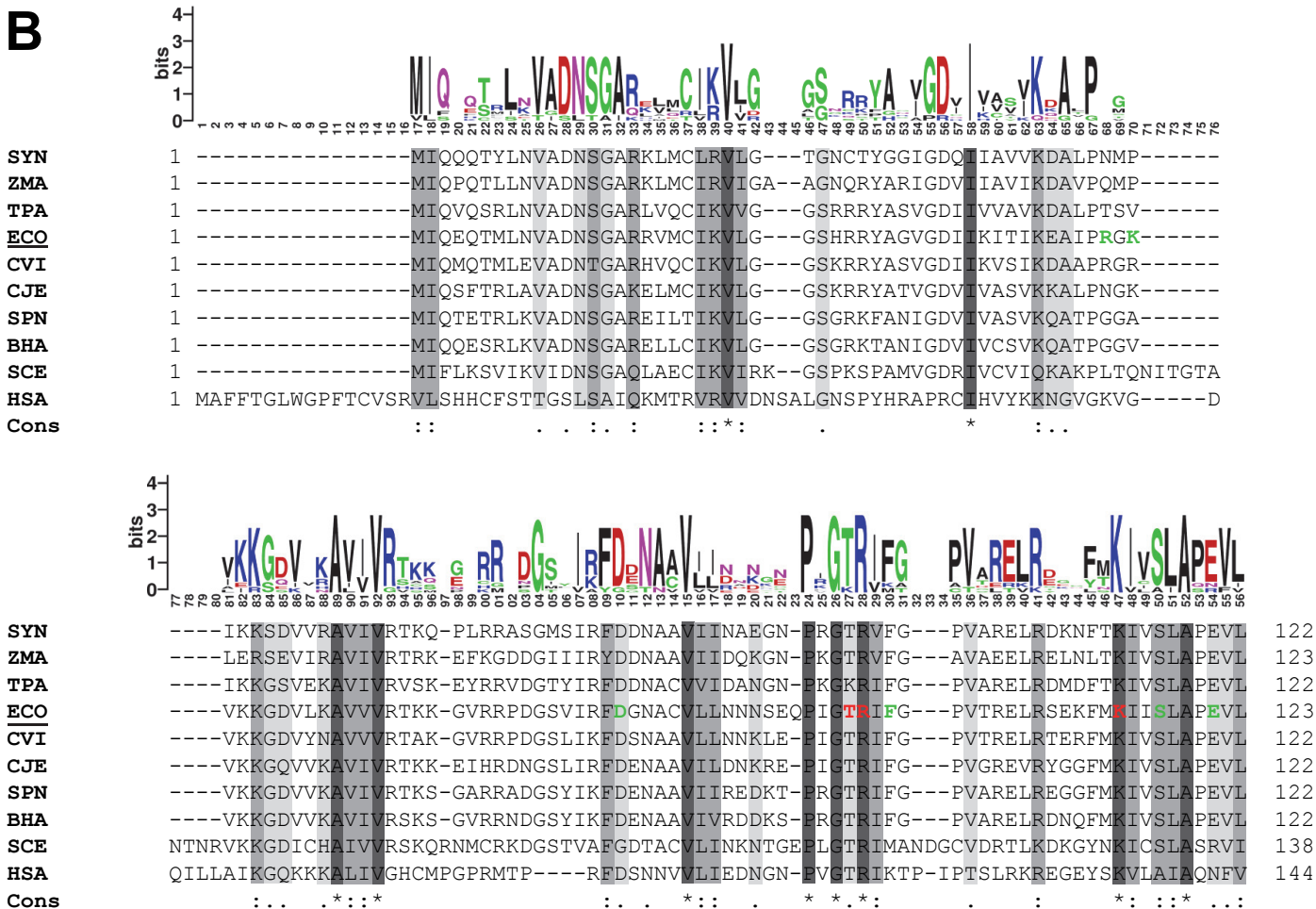

Supplement: Figure S1 — Multiple sequence alignments of selected RsfA and L14 orthologues. (A) Protein sequences of RsfA orthologues which were shown to interact with L14, as well as orthologues from yeast and two species with available 3D-structures (Chromobacterium violaceum, PDB id: 2ID1 and Bacillus halodurans, PDB id: 2O5A). (B) Multiple sequence alignment of corresponding L14 protein sequences (only plastidal or mitochondrial L14 are shown for Zea mays, human and yeast, respectively). Amino acid residues of E. coli L14 that have been exchanged to alanine for interaction epitope mapping (Figure 2B) are highlighted in red (residue is involved in RsfA binding) and green (not involved in RsfA binding). Numbers on the left and right of the alignment sequences indicate the alignment start and stop positions, respectively. Consensus sequences shown at the top of each alignment were constructed with WebLogo V2.8.2 using default settings [60]. Multiple alignments were made using ClustalW2 [47]. Abbreviations: SYN (Synechocystis sp. PCC 6803), ZMA (Zea mays), TPA (Treponema pallidum), ECO (Escherichia coli), CVI (Chromobacterium violaceum), CJE, (Campylobacter jejuni), SPN (Streptococcus pneumoniae), BHA (Bacillus halodurans), SCE, (Saccharomyces cerevisiae), HSA (Homo sapiens). (PDF) [file pgen.1002815.s002.pdf]

Fig. S2

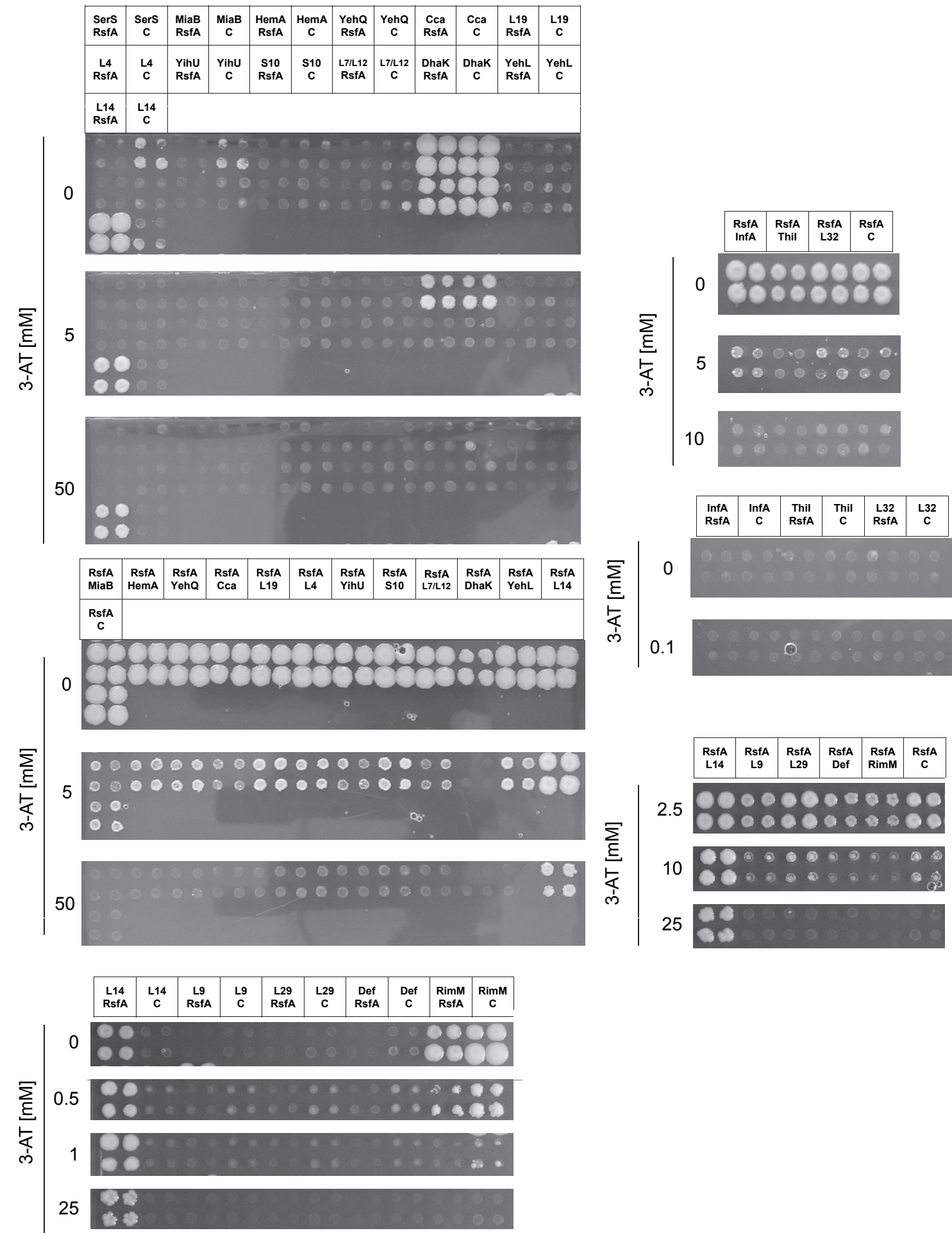

Supplement: Figure S2 — Interologue tests. Pairwise Y2H interaction assays carried out with homologous protein pairs of E. coli that have been detected for RsfA in other studies (tested interactions and reference sets, see Table S1). Protein pairs were tested reciprocally (i.e., RsfA tested as bait and prey fusion) as quadruplicates on various concentrations of 3-AT. Baits are shown on top, preys are below in the legends. “C”, negative control: bait constructs are tested against the prey vector that does not contain any insert to check for reporter gene self-activation of the bait. Only the E. coli interaction of RsfA with L14 turned out to be conserved. (PDF) [file pgen.1002815.s003.pdf]

Fig. S3

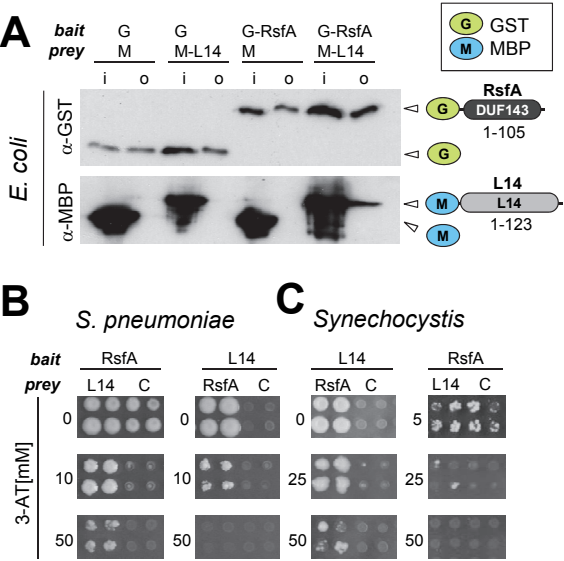

Supplement: Figure S3 — Interaction of RsfA-L14 in E. coli, S. pneumoniae, and Synechocystis. (A) Verification of E. coli RsfA-L14 interaction by a pull down assay. RsfA was tagged with glutathione S-transferase “G” and L14 with maltose binding protein “M”; i = input and o = output samples. (B, C) RsfA and L14 of Streptococcus pneumoniae TIGR4 (B) and Synechocystis PCC 6803 (C) interact in Y2H assays. Protein pairs were tested in quadruplicates on various concentrations of 3-AT. C, control (empty prey vector). (PDF) [file pgen.1002815.s004.pdf]
