# Supplementary material for: RsfA (YbeB) Proteins Are Conserved Ribosomal Silencing Factors
Source: PLoS Genet. 2012 Jul 19;8(7):e1002815. doi: 10.1371/journal.pgen.1002815 (PMC3400551; doi:10.1371/journal.pgen.1002815)
Supplement: Table S1 — RsfA and L14 and their interaction are conserved in bacteria and eukaryotic organelles. (A) Known interactions of RsfA with L14 orthologues and physical association with the LRS. The table summarizes all known binary interactions among RsfA-L14 orthologous pairs as well as co-purified ribosomal protein complexes from this and other studies. RsfA-L14 interactions identified by binary methods are highlighted in light grey. RsfA orthologues co-purified with protein complexes/the ribosome are highlighted in dark grey. Abbreviations used: LRS (large ribosomal subunit), Y2H (yeast-2-hybrid), MS (mass spectrometry), Co-IP (co-immunoprecipitation), BiFC (bimolecular fluorescence complementation). (DOC) [file pgen.1002815.s005.doc]

**Supporting Table S1. RsfA and L14 and their interaction are conserved in bacteria and eukaryotic organelles.** (**A**) Known interactions of RsfA with L14 orthologues and physical association with the LRS. The table summarizes all known binary interactions among RsfA-L14 orthologous pairs as well as co-purified ribosomal protein complexes from this and other studies. RsfA-L14 interactions identified by binary methods are highlighted in light grey. RsfA orthologues co-purified with protein complexes/the ribosome are highlighted in dark grey. Abbreviations used: LRS (large ribosomal subunit), Y2H (yeast-2-hybrid), MS (mass spectrometry), Co-IP (co-immunoprecipitation), BiFC (bimolecular fluorescence complementation).

| **Species** | **Orthologues**  **(locus tags)** | | **Method** | **Reference** |
| --- | --- | --- | --- | --- |
|  | **RsfA** | **L14/LRS** |  |  |
| *Escherichia coli* K12 | b0637 | 50S LRS | iTRAQ, pull down |  |
|  |  | b3310 (L14) | Y2H, pull down | this work |
|  |  |  | MS |  |
| *Treponema pallidum* | TP0738 | TP0199 (L14) | Y2H | , this work |
| *Campylobacter jejuni* | Cj1405 | Cj1697(L14) | Y2H |  |
| *S. pneumoniae* TIGR4 | SP1744 | SP0219 (L14) | Y2H | this work |
| *Synechocystis* PCC 6803 | slr1886 | sll1806 (L14) | Y2H | this work |
| *Saccharomyces cerevisiae* | YMR098C (=ATP25) | 54S LRS, mitochondrial | MS |  |
| *Homo sapiens* | C7orf30 | L14_mt_ (=MRPL14), mitochondrial | BiFC, pull down | this work |
|  |  | 39S LRS, mitochondrial | Co-sedimentation/IP |  |
| *Zea mays* | Ij (Iojap) | RPL14 (L14, chloroplastic) | Pull down | this work |
|  |  | 50S LRS, chloroplastic) | Co-IP |  |
