# Supplementary material for: RsfA (YbeB) Proteins Are Conserved Ribosomal Silencing Factors
Source: PLoS Genet. 2012 Jul 19;8(7):e1002815. doi: 10.1371/journal.pgen.1002815 (PMC3400551; doi:10.1371/journal.pgen.1002815)
Supplement: Table S2 — RsfA interactions tested negatively with E. coli homologous protein pairs in a Y2H experiment (see Supplementary Figure S2). Orthologues from T. pallidum and C. jejuni were selected by MBGD orthologous protein groups [61]. The reference set gives the source of RsfA orthologous interactions they were primarily described in. Note, the interaction partners identified by Butland et al. are proteins that have been co-purified as protein complex of E. coli RsfA. (DOC) [file pgen.1002815.s006.doc]

**Supporting Table S2. RsfA interactions tested negatively with *E. coli* homologous protein pairs in a Y2H experiment** (see **Supplementary Figure S2**). Orthologues from *T. pallidum* and *C. jejuni* were selected by MBGD orthologous protein groups . The reference set gives the source of RsfA orthologous interactions they were primarily described in. Note, the interaction partners identified by Butland et al. are proteins that have been co-purified as protein complex of *E. coli* RsfA.

| **Gene/protein name** | ***E. coli* locus tag** | **Reference species** | **Locus tag orthologue** | **Description (*E. coli* annotation by Uniprot )** | **Reference interaction set** |
| --- | --- | --- | --- | --- | --- |
| **MiaB** | b0661 | *C. jejuni* | Cj1454c | tRNA-i(6)A37 methylthiotransferase |  |
| **SerS** | b0893 |  | Cj0389 | Seryl-tRNA synthetase |  |
| **HemA** | b1210 |  | Cj0542 | Glutamyl-tRNA reductase |  |
| **S10** | b3321 |  | Cj1708c | 30S ribosomal protein S10 |  |
| **DhaK** | b1200 | *E. coli* | - | Dihydroxyacetone kinase, N-terminal domain |  |
| **YehL** | b2119 |  | - | Uncharacterized protein yehL |  |
| **YehQ** | b2122 |  | - | Predicted protein |  |
| **L19** | b2606 |  | - | 50S ribosomal subunit protein L19 |  |
| **Cca** | b3056 |  | - | Multifunctional CCA protein |  |
| **L4** | b3319 |  | - | 50S ribosomal protein L4 |  |
| **YihU** | b3882 |  | - | Uncharacterized oxidoreductase yihU |  |
| **L7/L12** | b3986 |  | - | 50S ribosomal subunit protein L7/L12 |  |
| **ThiI** | b0423 | *T. pallidum* | TP0559 | tRNA sulfurtransferase |  |
| **InfA** | b0884 |  | TP0097 | Translation initiation factor IF-1 |  |
| **L32** | b1089 |  | TP0807 | 50S ribosomal protein L32 |  |
| **RimM** | b2608 |  | TP0907 | Ribosome maturation factor rimM |  |
| **Def** | b3287 |  | TP0757 | Peptide deformylase |  |
| **L29** | b3312 |  | TP0197 | 50S ribosomal protein L29 |  |
| **L9** | b4203 |  | TP0060 | 50S ribosomal protein L9 |  |
